# The Search for Resistance to Cassava Mosaic Geminiviruses: How Much We Have Accomplished, and What Lies Ahead

**DOI:** 10.3389/fpls.2017.00408

**Published:** 2017-03-24

**Authors:** Vincent N. Fondong

**Affiliations:** Department of Biological Sciences, Delaware State UniversityDover, DE, USA

**Keywords:** cassava, geminiviruses, natural resistance, artificial miRNA, *trans*-acting siRNA, CRISPR

## Abstract

The cassava mosaic disease (CMD), which occurs in all cassava growing regions of Africa and the Indian subcontinent, is caused by cassava mosaic geminiviruses (CMGs). CMGs are considered to be the most damaging vector-borne plant pathogens. So far, the most successful approach used to control these viruses has been the transfer of a polygenic recessive resistance locus, designated CMD1, from wild cassava to cassava cultivars. Further progress in harnessing natural resistance to contain CMGs has come from the discovery of the dominant monogenic resistance locus, CMD2, in some West African cassava cultivars. CMD2 has been combined with CMD1 through genetic crosses. Because of the limitations of the cassava breeding approach, especially with regard to time required to produce a variety and the loss of preferred agronomic attributes, efforts have been directed toward the deployment of genetic engineering approaches. Most of these approaches have been centered on RNA silencing strategies, developed mainly in the model plant *Nicotiana benthamiana*. Early RNA silencing platforms assessed for CMG resistance have been use of viral genes for co-suppression, antisense suppression or for hairpin RNAs-mediated gene silencing. Here, progress and challenges in the deployment of these approaches in the control of CMGs are discussed. Novel functional genomics approaches with potential to overcome some of the drawbacks of the current strategies are also discussed.

## Introduction

**FIGURE 1:**
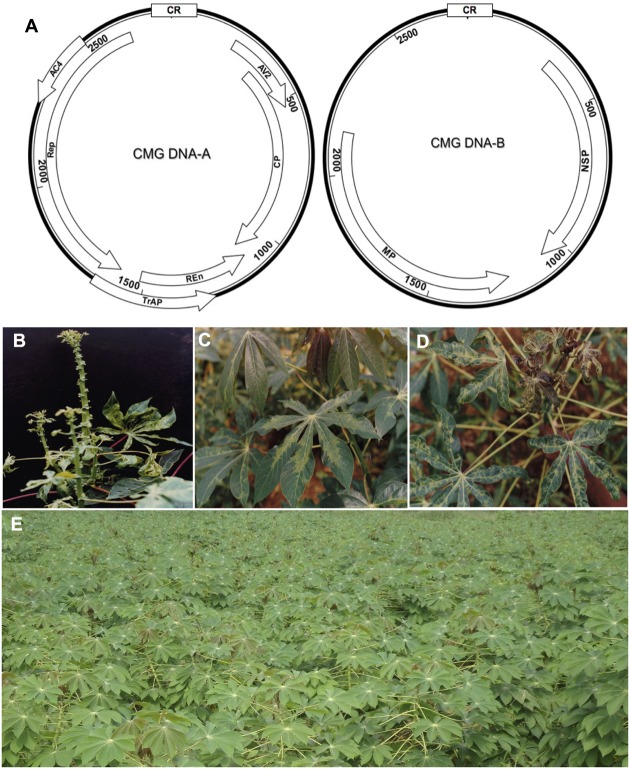
**Cassava mosaic geminiviruses (CMG) causal agents of the cassava mosaic disease (CMD). (A)** Genome organization of CMG. These viruses have two genomic components designated DNA-A and DNA-B. DNA-A has six genes, two in the virion-sense (*AV1* that codes for the coat protein, CP) and *AV2*; and four in the complementary-sense: *AC1* (codes for the replication associated protein, Rep), *AC2* (codes for transcription activation protein, TrAP), *AC3* (codes for replication enhancer protein, REn), and *AC4*. DNA-B codes for two genes: virion-sense *BV1* (codes for nuclear shuttle protein, NSP); and complementary-sense *BC1* (codes for the movement protein, MP). Except for an approximately 200-nt segment of the 5′ intergenic region (IR), designated the common region (CR), the two genomic components of each of these viruses are different in sequence. The CR contains an origin of replication (ori) and *cis* sequences required for viral DNA during replication. **(B)** Severe CMD symptoms in a highly susceptible cultivar infected by ACMV and EACMCV (Fondong et al., 2000a), **(C)** mild symptoms on TMS 8034 singly infected by ACMV, and **(D)** severe symptoms on TMS 8034 mixed infected by ACMV and EACMCV. **(E)** Field plot of the recently adopted highly resistant TMS 96/0023, which exhibits less than 2% CMD incidence in southwestern Cameroon.

Cassava, *Manihot esculenta* Crantz, was introduced in Africa by the Portuguese in the 16th century, and was initially grown in and around trading posts in the Gulf of Guinea in West Africa. It was subsequently introduced into East Africa from Madagascar in the later part of the 18th century (Legg and Thresh, 2000). Today, cassava provides staple food to an estimated 800 million people worldwide (FAO, 2013), and is grown almost exclusively by smallholder farmers in isolated areas where soils are poor and rainfall is low or unpredictable. Additional attributes of this crop include low-cost and readily available planting material, high tolerance to acid soils, and its symbiotic associations with soil fungi to help absorption of phosphorus and micronutrients. Thus, cassava production requires very few inputs and gives reasonable harvests where other crops would fail (FAO, 2013). However, production of this crop is severely limited by the cassava mosaic disease

(CMD), which is caused by cassava mosaic geminiviruses (CMGs, Family *Geminiviridae*: Genus *Begomovirus*) (Patil and Fauquet, 2009). CMGs are transmitted by the whitefly (*Bemisia tabaci*) or spread through infected cuttings, which are the usual mode of cassava propagation. The viral etiology of CMD was confirmed by electron microscopy of geminivirus particles (Bock and Woods, 1983). With the emergence of new molecular and sequencing techniques, CMGs with considerable sequence and biological differences continue to be identified across the African continent and in the Indian subcontinent, and 11 CMG species have been described (Patil and Fauquet, 2009; Legg et al., 2015) (**Table 1**). The genome organization of CMGs is shown in **Figure 1A**.

**Table 1:** Eleven cassava mosaic geminiviruses described to date.

CMG	Occurrence	Reference
*African cassava mosaic virus* (ACMV)	African continent	Bock and Woods, 1983; Stanley and Gay, 1983
*African cassava mosaic Burkina Faso virus* (ACMBFV)	Western Africa	Tiendrébéogo et al., 2012
*Cassava mosaic Madagascar virus* (CMMGV)	Madagascar	Harimalala et al., 2012
*East African cassava mosaic Cameroon virus* (EACMCV)	Eastern and Western Africa	Fondong et al., 1998
*East African cassava mosaic Kenya virus* (EACMKV)	Eastern Africa	Bull et al., 2006
*East African cassava mosaic Malawi virus* (EACMMV)	Eastern and Southern Africa	Zhou et al., 1998
*East African cassava mosaic virus* (EACMV)	Eastern Africa	Swanson and Harrison, 1994
*East African cassava mosaic Zanzibar Virus* (EACMZV)	Eastern Africa	Maruthi et al., 2004
*Indian cassava mosaic virus* (ICMV)	Indian Subcontinent	Hong et al., 1993
*South African cassava mosaic virus* (SACMV)	Southern Africa	Berrie et al., 1998
*Sri Lankan cassava mosaic virus* (SLCMV)	Indian Subcontinent	Saunders et al., 2002


Cassava mosaic disease is the most important disease of cassava in Africa; its symptoms typically include an irregular yellow or yellow–green chlorotic mosaic of leaves, leaf distortion, and stunted growth. Tuberous root losses due to CMD may reach 100% in highly susceptible varieties (Thresh et al., 1994; Masinde et al., 2016) or in mixed infections (Fondong et al., 2000a; Pita et al., 2001). Generally, there are three objectives to decreasing losses caused by virus diseases: (1) decrease the proportion of plants that become infected; (2) delay infection to such a late stage of crop growth that losses become unimportant; (3) decrease the severity of damage sustained after infection has occurred (Thresh and Cooter, 2005). These objectives can be achieved through phytosanitation (involving quarantine measures, crop hygiene, use of virus-free planting material and eradication), changes in cropping practices (e.g., intercropping), use of pesticides to control vectors, and deployment of resistant or tolerant varieties (Thresh, 2003). Of these control strategies, use of resistant varieties has been the most effective in controlling CMD in Africa. Thus, when in the 1930s it became apparent that some of the cassava varieties being grown were less affected by CMD than others, resistance breeding began in Ghana, Madagascar, Tanzania, and elsewhere in Africa (Thresh and Cooter, 2005). However, in the last two decades, use of genetic engineering to produce resistant cassava has gained considerable attention, especially with the discovery of RNA interference pathways at the end of the last century (Vanderschuren et al., 2007). The successes and failures of these CMG control approaches, as well as additional new opportunities offered by recent advances in genomics toward the containment of these viruses, are discussed in this review.

## CMG Resistance Breeding

In spite of the challenges with cassava breeding, the most successful CMD control intervention to date has been the introgression of resistance from wild cassava, *Manihot glaziovii* Muell.-Arg, into cultivated cassava. This gene transfer started in the 1930s following the CMD pandemics that occurred in eastern Africa in the 1920s when it became clear that *M. glaziovii* was resistant to CMD (Legg and Thresh, 2000). Thus, the Amani cassava breeding program in Tanzania carried out crosses between cassava germplasm and *M. glaziovii*. Hybrids from these crosses were backcrossed to cassava and the progeny obtained exhibited satisfactory root yield and quality (Jennings, 1994; Thresh et al., 1998). In 1971, seeds of resistant cassava genotypes were introduced to the International Institute of Tropical Agriculture (IITA) in Nigeria where crosses were made with local Nigerian varieties and South American germplasm from the International Center for Tropical Agriculture (CIAT) (Beck, 1982). Nigerian clonal selection 58308, which originated from the Tanzania breeding program, has since featured prominently as a parent in resistance breeding at IITA (Jennings, 1976; Beck, 1982; Nweke et al., 2002; Thresh and Cooter, 2005). Today, the IITA breeding program is the largest and most influential in Africa as it supplies parental material, seed and breeding lines to many national programs (Mahungu et al., 1994). Breeding efforts similar to, but independent from, the Tanzania program were conducted concurrently in Madagascar, where important local and introduced cultivars were crossed with *M. glaziovii*, followed by backcrossing with preferred cassava cultivars (Jennings, 1994).

The CMD resistance from *M. glaziovii*, designated CMD1, is today known to be polygenic (Akano et al., 2002). Consequently, the resistant cassava lines produced in breeding programs in Tanzania, Madagascar, IITA, and elsewhere in Africa have several important characteristics that are closely associated and are manifestations of the same basic virus resistance mechanism (Thresh et al., 1998). These include: (1) compared with existing local cultivars a lower proportion of plants become infected and the infected plants display less severe symptoms, (2) the virus is incompletely systemic, with symptoms being restricted to certain shoots or branches, and (3) resistant varieties contain lower concentrations of virus than do susceptible ones (Patil and Fauquet, 2015a; Kuria et al., 2016), as demonstrated serologically by Fargette et al. (1996). Because of low viral titers and the incomplete systemic spread of virus in these resistant varieties, a substantial proportion of the cuttings collected from infected plants are free of virus, even if taken from plants that were infected as cuttings or were infected at an early stage of growth by whiteflies. An important outcome of this effect is the so-called *reversion* phenomenon (Fargette et al., 1994; Fargette and Vid, 1995; Fondong et al., 2000b). *Reversion* is a phenomenon whereby cassava cuttings from virus-infected mother-plants, produce virus-free plants in the next cropping cycle, due to incomplete systemic spread of the causal virus (Fargette et al., 1994; Fondong et al., 2000b). A consequence of the high rate of *reversion* in IITA varieties is that they never become totally infected, even when grown for years in conditions of intense infection pressure, as observed in Cameroon (Fondong et al., 2000b) and Uganda (Gibson and Otim-Nape, 1997). Distribution of the resistant cassava developed at IITA began in early 1970. Thus, between 1970 and 1998, over 200 improved IITA varieties with CMD1 polygenic resistance were released in 20 African countries (Manyong et al., 2000). A clear advantage of adopting these resistant varieties is that farmers tend to discriminate in favor of vigorous and/or symptomless plants when selecting cuttings for new plantings. This significantly reduces virus spread through cuttings and whiteflies.

Building on the success of CMD1 polygenic resistance, IITA identified a monogenic CMD resistance locus, designated CMD2, in some West African cassava landraces (referred to as tropical *M. esculenta*, TME) (Akano et al., 2002). The two types of resistance are complementary in their effects, and have been combined by crossing TME and TMS (CMD1) parents to produce CMD3 (Lokko et al., 2001a,b). Several of the TME × TMS hybrids produced in the IITA breeding program have given very high yields, and carry additional desirable attributes of many landraces. Importantly, these progeny have tuberous roots of good taste and quality, and the plants have an erect growth habit that is preferred by many farmers. Several of the landraces and progeny derived from crosses with TMS clones are being grown in Nigeria and several other African countries, including Cameroon, Kenya, Madagascar, Rwanda, Tanzania, and Uganda. Indeed, today, National Agricultural Research programs in 31 African countries have received improved CMD resistant germplasm from the IITA breeding program (Alene et al., 2015). A clear additional advantageous attribute of the IITA varieties is that they have a level of resistance or tolerance to other major pests of cassava, such as bacterial blight disease, anthracnose disease, cassava green mite, and cassava mealybug (Dixon et al., 2010). The successes recorded with these two sources of CMD resistance have led to use of marker-assisted selection in South America to introduce the CMD1 and CMD2 genes to local populations as a precautionary measure against the real possibility of CMD appearing in the South American continent (Thresh and Cooter, 2005; Okogbenin et al., 2012).

In several countries, varieties selected from the IITA germplasm have been shown to exhibit strong resistance to CMD. For example, in Uganda, where the latest CMD pandemic first emerged in the 1990s, the IITA varieties were resistant to CMD, and to a lesser extent, cassava brown streak disease (CBSD), caused by cassava brown streak viruses (CBSVs) in East Africa (Ntawuruhunga et al., 2013; Pariyo et al., 2015; Patil et al., 2015). Similar successes have been recorded in other eastern and southern African countries (Ntawuruhunga et al., 2013; Mbewe et al., 2015; Masinde et al., 2016). In Zambia, eight IITA varieties outperformed local varieties significantly, resulting in increased yields, household income, and food security (Khonje et al., 2015). As for the varieties selected in the Democratic Republic of Congo (DRC), CMD incidence was 15% in IITA varieties compared with 100% incidence in local varieties in locations assessed (Muengula-Manyi et al., 2012). In the case of Cameroon where IITA germplasm was first introduced in the 1980s, three adopted varieties proved to be very popular: TMS 8017, TMS 8034, and TMS 8061. Unlike local varieties that display severe symptoms, especially when mixed infected (**Figure 1B**), all three IITA varieties recorded CMD incidences of less than 20% in high infection pressure areas in the southwestern part of the country (Fondong, 1999). In all three varieties, infected plants tended to display mild symptoms except when doubly infected by ACMV and EACMCV (**Figures 1C,D**). Since 1999, five new IITA cassava varieties containing both CMD1 and CMD2 resistances have been adopted and shown to display CMD incidences of less than 10% in the high inoculum pressure areas of southwestern Cameroon where local cultivars show 100% incidence; indeed, one of these varieties, TMS 96/0023 (**Figure 1E**), displays a strong resistance to CMD.

## Reversion

An understudied mechanism of CMG resistance in cassava is the *reversion* phenomenon, which is the ability of cuttings from CMG infected mother-plants to grow into virus-free plants in the next cropping cycle. This mechanism has been attributed to incomplete systemic spread of the causal virus in infected plants (Fargette et al., 1994; Fondong et al., 2000b). It was first reported in cassava by Storey and Nichols (1938) and is known to play a crucial role in containing the spread of CMD epidemics (Gibson and Otim-Nape, 1997; Fondong et al., 2000b). Interestingly, *reversion* has also been reported in CBSV-infected cassava (Mohammed, 2012). It has also been reported in other clonally propagated plant species, including sweet potato infected by *Sweet potato feathery mottle virus* (SPFMV) and *Sweet potato chlorotic stunt virus* (SPCSV) (Gibson et al., 1997); potato infected by *Potato leafroll luteovirus* (Bertschinger, 1990); subterranean clover infected by *Subterranean clover mottle sobemovirus* (Wroth and Jones, 1992) and strawberry infected with *Strawberry crinkle cytorhabdovirus* and *Strawberry mottle sadwavirus* (Frazier et al., 1965). Despite the importance of this phenomenon in countering virus epidemics, we know very little about the molecular basis of its occurrence.

*Reversion* from CMD has been shown to occur at different rates in different cassava genotypes under both field and controlled environment conditions in Cameroon (Fondong et al., 2000b), Uganda (Gibson and Otim-Nape, 1997), and Cote d’Ivoire (Fargette et al., 1994). This phenomenon has been linked to recovery, which is the ability of originally symptomatic plants to partially or completely lose symptoms in the upper stem (Gibson and Otim-Nape, 1997). This link is consistent with field reports by Fondong et al. (2000b) that show that cuttings obtained from upper portions of the stem produce higher rates of *reversion* than do those from the lower stem. Further evidence of a link between recovery and *reversion* has come from reports that under high temperature conditions, CMD infected plants exhibit a high rate of recovery (Chellappan et al., 2005; Patil and Fauquet, 2015a,b), and this is consistent with observations under field conditions showing a high rate of reversion at low altitude locations where average temperatures are high compared with high altitude sites with low temperatures (Fondong et al., 2000b). A further confounding phenomenon of epidemiological importance is the effect of double infections on *reversion*. This is because mixed infections of the same plant by ACMV and EACMCV are expected to display low *reversion* rates, given that mixed infected plants display more severe symptoms than do singly infected plants (Fondong et al., 2000a; Vanitharani et al., 2004) and do not recover from infection, as has been observed for TMS 8017 in Cameroon (**Figure 1D**).

Recently, it was reported that CMD-susceptible cassava cultivars that displayed no recovery or showed a weak recovery, accumulate higher levels of viral small interfering RNAs (vsiRNAs) compared with genotypes that exhibited a strong recovery (Bengyella et al., 2015; Patil and Fauquet, 2015a; Kuria et al., 2016; Rogans et al., 2016). While these observations provide the first insights into small RNA (sRNA) profiles of CMD recovered and non-recovered phenotypes, important questions remain. For example, it is possible that differences in vsiRNA levels recorded in the studies were at least partially due to genetic variation between the genotypes analyzed. Also, both studies examined only vsiRNA, yet it is likely that there are different expression levels of host sRNAs derived from host genes involved in the recovery phenotype. Furthermore, in accordance with evidence linking methylation with recovery from geminivirus infection (Raja et al., 2008, 2014), the Rogans et al. (2016) study suggested a possible role for methylation in host recovery from CMD, based on the levels of 24-nt siRNAs, which in the canonical RNA-directed DNA methylation (RdDM) pathway direct ARGONAUTE 4 (AGO4)-mediated methylation of targets (Xie and Yu, 2015). It will therefore be important to determine the methylation state of host and viral genomes, as well as identify additional interacting proteins involved. Uncovering all factors involved in the recovery phenotype will provide new opportunities in understanding this type of host resistance. It will also clarify the possible link between the recovery phenotype and *reversion* and allow the identification of markers that can be used to select high reverting cultivars as this would have a direct and immediate impact to farmers, especially given field observations by the author that reverted plants tend to be more resistant to infection by whitefly vectors.

## Engineered Resistance to CMGs

The ability of genetically engineered plants to express viral genes to be cross-protected from future infection by the cognate virus was first elucidated in plants expressing the coat protein (CP) genes of *Tobacco mosaic tobamovirus* and *Alfalfa mosaic alfamovirus*, respectively (Baulcombe et al., 1986; Powell-Abel et al., 1986). Subsequently, it was shown that plants stably expressing *Cucumber mosaic cucumovirus* (CMV) satellite RNA were protected from CMV (Tumer et al., 1987). The ability of genome segments of a pathogen to confer resistance to subsequent infection by the same pathogen was appropriately designated pathogen-derived resistance (PDR), and has been used to generate resistances to a broad range of viruses (reviewed in Gottula and Fuchs, 2009 and in Collinge et al., 2010). Early efforts aimed at explaining these types of PDR suggested at least three mechanisms: (1) competition between the transgene and virus, (2) prevention of virus uncoating by the transgene, and (3) that there was RNA-RNA annealing between the transgene and viral RNA (Nelson et al., 1987) leading to co-suppression of both genes (Jorgensen, 1990). Intriguingly, in the geminiviruses, neither transgenic beans expressing the CP gene of *Bean golden mosaic begomovirus* (BGMV) (Azzam et al., 1996) nor *Nicotiana benthamiana* plants expressing ACMV CP (Frischmuth and Stanley, 1998) displayed any resistance to the respective virus. Further, the occasional resistance of *Tomato mottle begomovirus* (TMoV)-CP transgenic tobacco plants to TMoV was not correlated with CP expression (Sinisterra et al., 1999); similar observations were reported for *Tomato leaf curl virus* (TLCV) (Selth et al., 2004). Although, mechanistically the failure of the *CP* to confer resistance in transgenic plants has not been explained, this may at least partially be due to the fact that the *CP* is a late gene and not required for virus replication. Thus, viral DNA could replicate and spread without an accumulation of the *CP* gene. However, other geminivirus genes and genome segments were found to confer resistance and this approach has been used extensively to develop resistances to numerous geminiviruses, including CMGs (reviewed in Vanderschuren et al., 2007). For CMGs, these technologies have been assessed mainly in *N. benthamiana*, principally due to the difficulty of transforming cassava, which is recalcitrant to genetic transformation (Li et al., 1996). These PDR approaches and the outcomes with regards to countering CMGs are described below.

### Sense, Antisense, and Inverted Repeat Transgenes

The inability of the CPs of ACMV and BGMV to confer PDR resistance (Frischmuth and Stanley, 1998; Sinisterra et al., 1999) led CMG researchers to assess other viral genes for the ability to confer a resistance. The first obvious choice was *AC1*, which is the only geminivirus gene that is indispensable for viral replication. Thus, Hong and Stanley (1996) generated transgenic *N. benthamiana* containing the *AC1* gene from the Kenyan isolate of ACMV (ACMV-KE) under the control of *Cauliflower mosaic virus* (CaMV) 35S promoter. The resulting transgenic lines showed resistance to ACMV, characterized by low levels of viral DNA accumulation compared with control plants. Correspondingly, transgenic *N. benthamiana* plants expressing ACMV-KE *AC1* with a mutation at the nucleoside triphosphate (NTP)-binding site exhibited a delay in symptom appearance and some of the plants also displayed attenuated symptoms (Sangare et al., 1999). As expected, resistant plants accumulated lower levels of viral DNA compared with non-transgenic control plants and the resistance was shown to correlate with expression levels of the *AC1* transgene. Further studies showed that a short segment from *AC1* that codes for 57 N-terminal amino acids of the ACMV Rep protein was sufficient to inhibit ACMV DNA replication, similar to observations made in plants containing the full-length *AC1* (Hong and Stanley, 1995, 1996). Mechanistically, this resistance was attributed to a disruption of *AC1* transcription from the infecting virus and/or induction of instability of the viral *AC1* transcript (Sangare et al., 1999). It was also suggested that transgenic *AC1* interfered with virus replication by interacting with regulatory sequences located upstream of the *AC1* gene in the viral genome (Norris et al., 1996).

**FIGURE 2:**
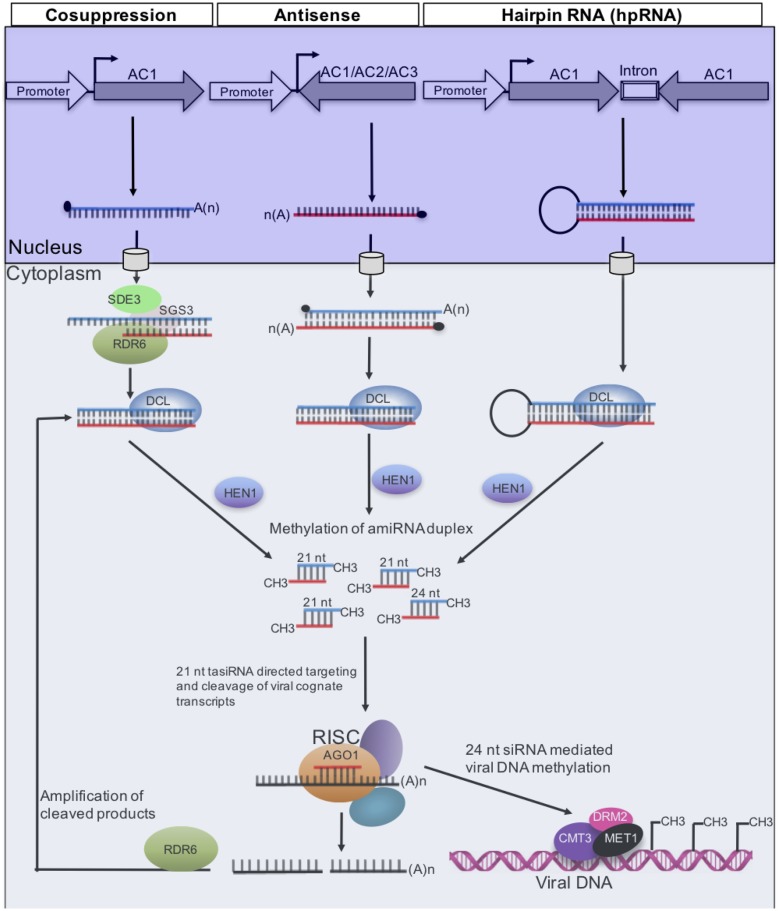
**Gene silencing pathways that have been used to generate resistance to CMGs.** Long dsRNAs generated from ACMV-KE *AC1* (sense) by a member of the RNA-dependent RNA polymerase (RDR) family, or antisense *AC1*, which binds to *AC1* transcripts, or *AC1* inverted repeat. In each case, the resulting dsRNA is processed by DCL4 into 21- and 24-nucleotide siRNAs duplexes, which are 2′ O-methylated at the 3′ end by HEN1 prior to entry into the RNA-induced silencing complex (RISC) where there is duplex unwinding. Only one strand of the siRNA duplex associates with the AGO effector protein (Tomari and Zamore, 2005). This guide strand directs to AC1 target by Watson-Crick base pairing; the second siRNA strand is degraded. Methylation of viral DNA is guided by 24-nucleotide siRNAs, a process mediated by DNA methyltransferases MET1, CMT3, and DRM2 (Raja et al., 2008). RNA polymerase RDR6, putative RNA helicase SDE3, coiled-coil protein SGS3, and AGO1 are required for sense transgene, but not for hpRNA PTGS (Wassenegger and Krczal, 2006; Diaz-Pendon et al., 2007).

The encouraging results recorded in *AC1* transgenic *N. benthamiana* led to the transfer of constructs to cassava, principally the West African cultivar, TMS60444, which was first transformed with ACMV-LE *AC1* under the control of the *Cassava vein mosaic virus* (CsVMV) promoter (Verdaguer et al., 1996). Under greenhouse conditions, these transgenic TMS60444 lines were observed to exhibit resistance to ACMV. Similar observations were reported by Chellappan et al. (2004), who further showed that transgenic TMS60444 lines expressing ACMV *AC1* were resistant, not only to ACMV but also to EACMCV and *Sri Lanka cassava mosaic begomovirus* (SLCMV). By the time, it had become clear that post transcriptional gene silencing (PTGS), a natural component of plant defense against viral infection mediated by siRNAs, was responsible for the resistance generated from a viral transgene (Hamilton and Baulcombe, 1999; Waterhouse et al., 2001). Thus, *AC1*-derived siRNAs were observed to accumulate in transgenic plants prior to virus inoculation (Chellappan et al., 2004). The fact that plants expressing ACMV *AC1* conferred resistance to ACMV, EACMCV, and SLCMV was therefore explained by sequence homology between the siRNAs and transcripts of all three CMGs. Subsequent studies further showed that TMS60444 transgenic lines expressing antisense strands of *AC1*, *AC2*, and *AC3* genes, exhibited resistance to ACMV, characterized by significantly reduced viral DNA levels (Zhang et al., 2005). Consistent with the results of Chellappan et al. (2004), and in agreement with the PTGS mechanism as illustrated in **Figure 2**, both antisense and sense siRNAs corresponding to respective genes accumulated in transgenic plants. These results are supported by a recent study (Patil et al., 2016) showing that transient expression in *N. benthamiana* of PTGS constructs targeting ACMV-CM *AC1*, *AC2*, and *AC4* generated ACMV resistance, characterized by production of high levels of siRNA especially along *AC2*.

In 1998, it was reported that transforming plants with viral gene constructs that produce RNAs capable of duplex formation (or hairpin RNA, hpRNA), conferred virus immunity (Waterhouse et al., 1998). This resistance was shown to be stronger than that generated through co-suppression or of antisense suppression. Therefore, Vanderschuren et al. (2009) expressed a 155-nucleotide segment of ACMV-KE *AC1* in the cassava cultivar TMS60444 as a hairpin double-stranded RNA (dsRNA) under the control of the CaMV 35S promoter. Consistent with a recent transient expression study (Patil et al., 2016), transgenic cassava lines with high levels of AC1 siRNAs were observed to display immunity to ACMV, and levels of siRNAs were correlated with ACMV resistance. Correspondingly, a hpRNA construct containing the overlapping region between SLCMV *AV1* and *AV2* was recently used to transform cultivar KU50, which is cultivated extensively in Southeast Asia for fuel production (Ntui et al., 2015). Transgenic lines obtained displayed high levels of resistance to SLCMV compared with wild-type plants, and PCR analyses failed to detect viral DNA in systemic uninoculated leaves, suggesting immunity to SLCMV.

Although not commonly assessed for their ability to confer resistance, the B component of geminiviruses have also been shown to induce host resistance. *N. benthamiana* stably expressing *Tomato golden mosaic begomovirus* MP inhibited ACMV replication (Von Arnim and Stanley, 1992). Similarly, tobacco plants expressing a mutated version of TMoV MP also showed resistance to TMoV and *Cabbage leaf curl begomovirus* (CaLCV) infection (Duan et al., 1997).

### ACMV Defective Interfering DNA

Prior to engineering plants for ACMV resistance using viral genes, Stanley et al. (1990) had shown that *N. benthamiana* transformed with an ACMV-derived defective interfering (DI) DNA displayed ACMV resistance. The DI DNA in this case was ACMV DNA B component from which large segments had been deleted, including BV1, but which retained the CR and parts of BC1 (Stanley and Townsend, 1985). Similar results were subsequently reported for corresponding DIs from SLCMV (Patil et al., 2007). The transgenic plants displayed symptom amelioration characterized by increased levels of subgenomic DNA and a comparable reduction in the level of genomic DNA. The greatest reduction was recorded in DNA B, suggesting that the DI DNA interfered especially with the replication and accumulation of DNA-B component. The fact that these plants did not display resistance to *Beet curly top virus* (BCTV) or TGMV indicated that the resistance against ACMV was specific. This resistance was explained by preferential replication of the episomal DI DNA at the expense of the viral genomic components. Thus, the infecting ACMV was able to mobilize the DI DNA from the *N. benthamiana* genome and to replicate it episomally. This result was in agreement with previous transient expression studies where *N. benthamiana* plants co-inoculated with ACMV-KE and the DIs resulted in reductions in replication of both ACMV-KE DNA A and B components (Etessami et al., 1988). Because of the specificity of this resistance, it became immediately clear that this approach could not be used to generate resistance to other CMGs because of differences between their genome sequences.

### NON-viral Transgenes

Several non-viral genes have been investigated for their ability to induce host resistance to geminiviruses, including CMGs (reviewed in Vanderschuren et al., 2007). For instance, dianthin, a ribosome inactivating protein with plant cytotoxic properties from *Dianthus caryophyllus*, was used to generate resistance to ACMV in *N. benthamiana*. In this case, to avoid generalized cell death, the gene was expressed under the control of the virion-sense promoter that is transactivated by TrAP and thus dianthin could only be activated by ACMV infected cells expressing TrAP (Hong et al., 1996), leading to death of only infected cells. As expected, transgenic plants produced necrotic lesions on inoculated leaves and showed significantly lower viral DNA levels. The failure of these plants to exhibit resistance to TGMV was consistent with the specificity of the interaction. In another instance, single stranded DNA (ssDNA)-binding proteins were assessed for their ability to control CMGs as previously elucidated in other geminiviruses (Padidam et al., 1999; Sera, 2005). Thus, the *Agrobacterium* Ti plasmid virulence gene *virE2*, encoding a nuclear ssDNA binding protein was expressed in *N. benthamiana.* The resulting transgenic plants were resistant to SLCMV (Resmi et al., 2015), in agreement with a report by Sunitha et al. (2011) showing *virE2*-mediated resistance to *Mungbean yellow mosaic begomovirus* (MYMV).

## New Genomic Approaches in the Control of CMGs

The encouraging early results obtained from the use of hpRNA to control plant viruses have been tempered by several disadvantages associated with hpRNA-induced resistance, these include poor stability of the transgene in transformed plants, dependence on the expression levels of the antisense strand, and limited penetration of the silencing signal to the appropriate viral target due to target-sequence folding (reviewed in Fondong et al., 2016). Furthermore, because whole viral genes or long genome segments had been used in most hpRNA studies, there was the risk of recombination between the transgenes and the infecting virus genomes, leading to emergence of new viruses (Morroni et al., 2009; Tepfer et al., 2015). Indeed, Frischmuth and Stanley (1998) reported a recombination between an ACMV *AV1* transgene in *N. benthamiana* and an agroinoculated ACMV DNA with a deleted *AV1* gene, resulting in ACMV with a functional CP. The development of new strategies to control CMGs is therefore imperative. To this end, recent advances in genomics have resulted in the discovery of new pathways that are being explored for use in crop improvement. Here, microRNA (miRNA), *trans*-acting small interfering RNA (tasiRNA) and Clustered Regularly Interspaced Short Palindromic Repeats associated Cas9 (CRISPR-Cas9) mechanisms and their application in generating resistance to CMGs are discussed.

### Artificial MicroRNAs (miRNAs)

MicroRNAs are endogenous sRNAs that play important regulatory roles in animals and plants by targeting mRNAs for cleavage or translational repression in a homology-dependent manner (Hutvagner and Zamore, 2002). miRNA biogenesis starts with the transcription of long primary RNAs (pri-miRNAs), which form stem-loop structures consisting of a terminal loop. An RNase III-type endonuclease, Dicer-like 1 (DCL1), processes the pri-miRNA to precursor miRNAs (pre-miRNAs) and in turn to a miRNA duplex. To silence genes, one strand of the duplex, the mature miRNA, is loaded onto an AGORNAUTE (AGO) protein, while the other strand, the miRNA^∗^, seems to be preferentially degraded (Hutvagner and Zamore, 2002; Bartel, 2004). AGO is the core protein of the RNA-induced silencing complex (RISC) that executes the silencing reaction.

**FIGURE 3:**
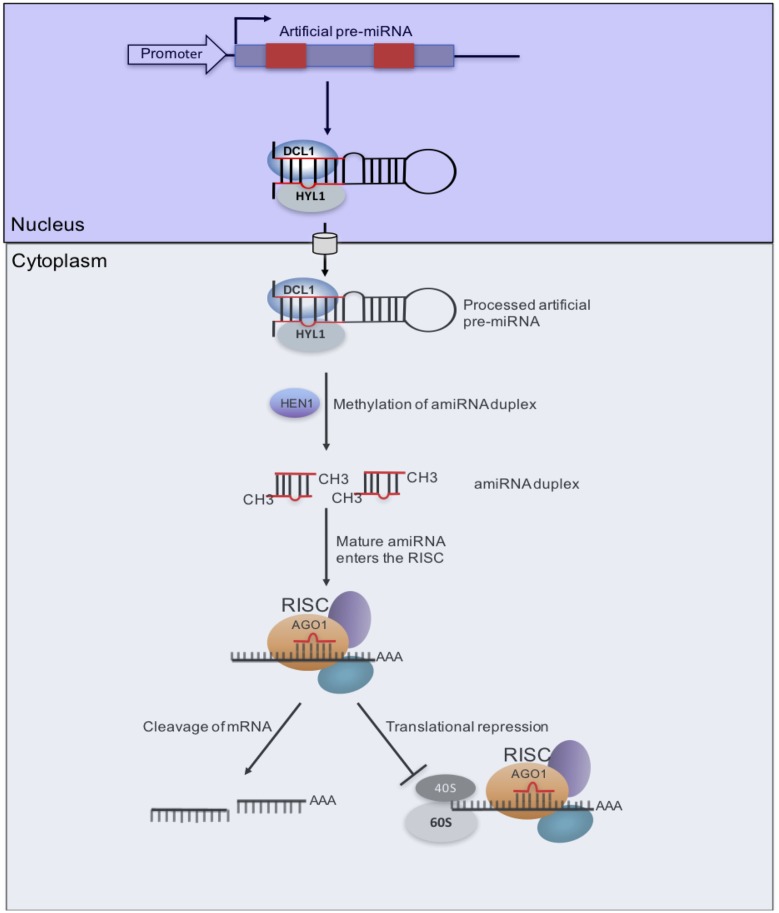
**Artificial miRNA (amiRNA) pathway for virus resistance. amiRNA/amiRNA^∗^s (red box) designed from a CMG genome is inserted into a pre-miRNA backbone.** After transcription, the pre-miRNA transcript folds back as a hairpin structure. The combined nuclear action of DCL1 and Hyponastic Leaves1 (HYL1) produces an amiRNA/amiRNA^∗^ duplex, which is methylated by HEN1. Upon nuclear export, the mature amiRNA is incorporated into AGO1-loaded RISC to promote two possible outcomes that are not mutually exclusive. A first process (left) would lead to endonucleolytic cleavage of homologous RNA, as directed by the viral amiRNA, which can also direct inhibition of translation (right), possibly at the initiation level (Brodersen and Voinnet, 2006).

Replacing the endogenous miRNA duplex within the natural miRNA precursor does not affect its biogenesis, as long as the secondary structure of the pre-miRNA is maintained (Ossowski et al., 2008). Therefore, the endogenous miRNA duplex can be replaced with an artificial sequence designed from genes to be targeted and silenced to generate an artificial miRNA (amiRNA)-miRNA^∗^ duplex. Upon processing, the amiRNA redirects the miRNA-induced silencing complex to silence the cognate target, thereby generating a loss-of-function phenotype for the target gene (Parizotto et al., 2004; Alvarez et al., 2006; Schwab et al., 2006; Ossowski et al., 2008; Wagaba et al., 2016) (**Figure 3**). So far, resistance to at least 12 plant viruses (including CBSV) has been generated using amiRNAs (see review in Fondong et al., 2016). This platform has great potential to generate CMG resistance because unlike hpRNAs, amiRNAs are small (21-nt) and thus the likelihood of off-target effects is reduced considerably. Also, the small size of amiRNAs makes it unlikely that there would be recombination with related viruses leading to the emergence of new viruses (Fondong et al., 2016). Moreover, this approach can be multiplexed by replacing miRNAs in a polycistronic miRNA backbone with different amiRNAs designed from different viruses to target cognate viruses. Indeed, a multiplex amiRNA in which five miR395s in the polycistronic miRNA backbone were replaced with amiRNAs from different *Wheat streak mosaic tritimovirus* (WSMV) genome regions conferred immunity to WSMV in transgenic plants (Fahim et al., 2012). Similarly, the polycistronic precursor of miR171 was used to produce three amiRNAs that targeted conserved segments in the *Wheat dwarf mastrevirus* (WDV) genome (Kis et al., 2016). Even though amiRNAs-derived resistance has not been reported in begomoviruses, it is likely that this approach would generate resistance in these viruses, especially if the amiRNA is designed to target the *Rep* gene, which is the only gene indispensable for viral replication.

The encouraging results of multiplex amiRNAs offer an unprecedented opportunity in the control of CMGs, which exhibit considerable genetic variability and frequently co-infect the same plant (Fondong et al., 2000a; Pita et al., 2001). Therefore, a multimeric amiRNA construct in which multiple amiRNAs targeting different conserved regions of different CMGs can be mounted on the same backbone of a polycistronic miRNA to simultaneously target cognate viruses. Use of multiplex amiRNA targeting multiple conserved regions also addresses the limitation that the small size of amiRNAs could create opportunities for the virus to evolve and escape surveillance due to loss of complementarity with amiRNAs (Lin et al., 2009).

### *Trans*-Acting Small Interfering RNA (tasiRNA)

**FIGURE 4:**
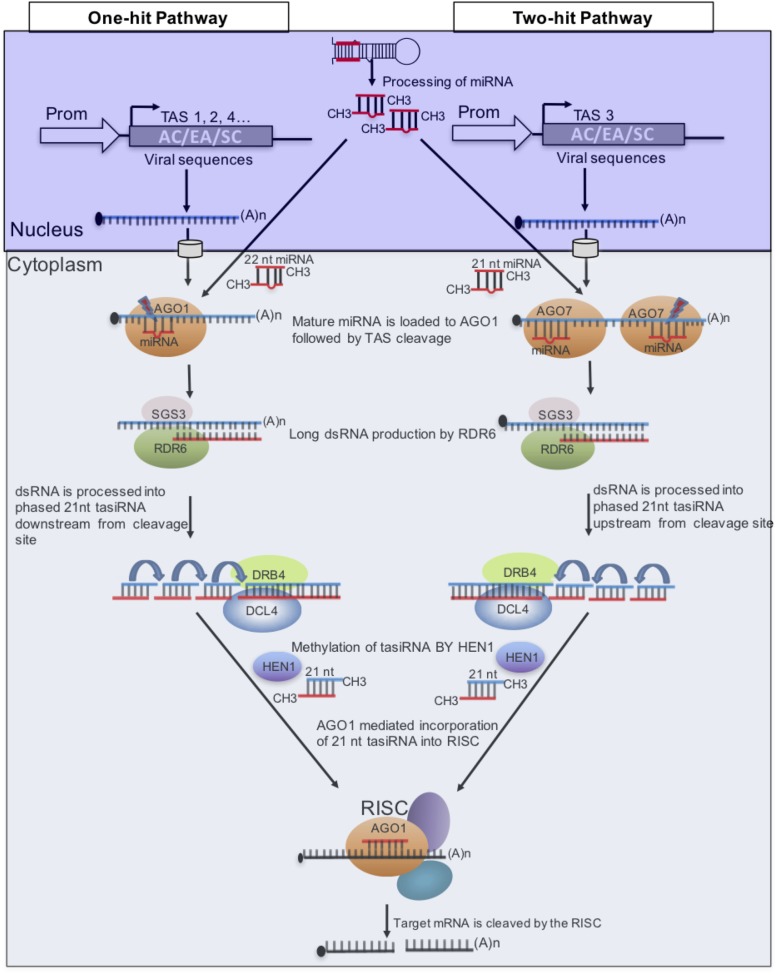
**Model of microRNA (miRNA) triggers and target sites of plant *trans*-acting siRNA (tasiRNA) biogenesis.** Two pathways are known to generate tasiRNAs in plants. To target three CMGs, sequences of ACMV (AC), EACMCV (EA), and SACMV (SC) are placed downstream of the miRNA-binding site in the “one-hit” model and between the two binding sites in the “two-hit” model. This strategy can also be used to target different regions of a viral genome to increase efficiency.

In plants, certain miRNAs induce the production of tasiRNAs from *trans*-acting siRNA (*TAS*) transcripts following an initial cleavage of the transcript by a RISC containing AGO1 or AGO7, depending on the specific miRNA. Two models of tasiRNA biogenesis referred to as “one-hit” and “two-hit,” respectively, have been described (Fei et al., 2013), (**Figure 4**). In the “one-hit” model, where there is a single miRNA target site, a RISC containing AGO1-bound 22-nt miRNA, binds and cleaves the *TAS* transcript, under the control of AGO1 (Chen et al., 2010; Cuperus et al., 2010). As for the “two-hit” model, two miRNA binding sites are used; however, 21-nt miRNA-directed AGO7 cleavage of the target occurs only on the 3′ binding site and the 5′ site is required but not cleaved (Axtell et al., 2006). In both models SGS3 stabilizes cleavage products that are processed into dsRNA by RDR6. DCL4 then cleaves the dsRNA products into phased 21-nt dsRNA registers downstream from the cleavage site for the “one-hit” model and upstream from the 3′ cleavage site for the “two-hit” model (Allen and Howell, 2010). These tasiRNA duplexes are methylated by HUA ENHANCER1 (HEN1) and only one strand of the duplex targets, *in trans*, the corresponding mRNA for cleavage, thereby silencing it.

Given that a single *TAS* transcript produces multiple tasiRNAs in a phased manner, *TAS* genes have been engineered to express multiple artificial tasiRNAs that target multiple viruses at several distinct genomic positions. In this approach therefore, the *TAS* gene is modified to contain viral sequences downstream of the miRNA cleavage site for the “one-hit” model or upstream of the cleavage site for the “two-hit” model (**Figure 4**). This strategy has been used to successfully engineer resistance to plant viruses. For example, transgenic *N. benthamiana* harboring a *TAS3* gene modified to contain tasiRNA from the *AC2* and *AC4* genes of *Tomato leaf curl New Delhi begomovirus* (ToLCNDV) exhibited strong resistance to ToLCNDV and to *Tomato leaf curl Gujarat begomovirus* (ToLCGV) (Singh et al., 2015). Also, transgenic *Arabidopsis thaliana* plants expressing *TAS3* modified with tasiRNAs targeting *Turnip mosaic potyvirus* and CMV were highly resistant to both viruses (Chen et al., 2016). Thus, tasiRNAs designed to target CMGs will likely provide a strong resistance to CMD.

There are many advantages of using the tasiRNA approach to control viruses. Importantly, unlike amiRNAs that requires a polycistronic miRNA pre-miRNA backbone, simply mounting multiple 21-nucleotide sequences adjacent to the miRNA binding site can multiplex tasiRNAs. Also, tasiRNAs are processed from ssRNA transcripts and thus there are no considerations for proper folding into dsRNA as found in amiRNA and hpRNA approaches. Furthermore, the systemic spread of tasiRNA throughout a plant is very efficient compared with sRNAs of other PTGS approaches (Felippes and Weigel, 2009). Finally, tasiRNAs selectively silence target genes without toxicity or off-target silencing, and recombination of transgenes with related viruses resulting in emergence of more virulent viruses is unlikely to occur due to the small size of tasiRNAs. The main disadvantage of the tasiRNA strategy is that it requires co-expression of exogenous miRNAs, *TAS* genes and their promoters; this is principally because current applications use well-studied *Arabidopsis TAS* genes. However, we recently identified endogenous cassava *TAS* loci and their miRNA triggers (Khatabi et al., 2016), which can be harnessed to express sequences of targeted viruses in cassava. In such a scenario, only viral sequences are inserted into the *TAS* gene.

Taken together, because sequences as small as 21-nt can be used in the tasiRNA approach, this platform provides an unprecedented opportunity to generate resistance to multiple CMGs from one construct given that sequences as long as 500 nucleotides were efficiently processed into 21-nt tasiRNAs (de la Luz Gutiérrez-Nava et al., 2008). Therefore, multiple 21-mers from different CMGs would be mounted into a *TAS* gene, which would then be processed into pools of tasiRNAs to target cognate viral sequences, thereby controlling the viruses.

### Clustered Regularly Interspaced Short Palindromic Repeats (CRISPRs)

**FIGURE 5:**
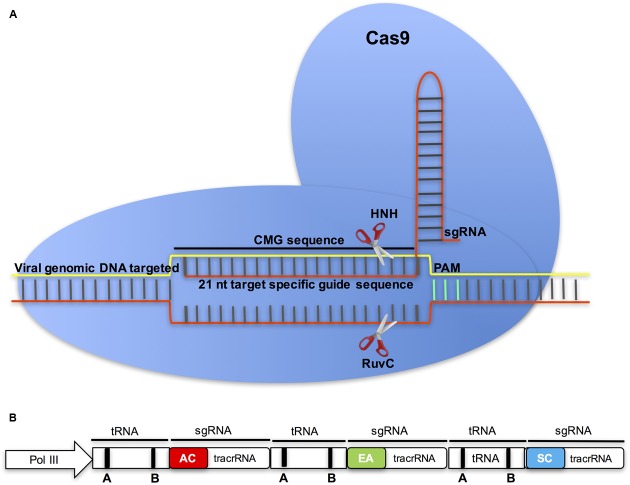
**The Cas9/sgRNA endonuclease in CMG control. (A)** To target CMGs, the ∼21-nt protospacer of the sgRNA is designed to target the viral sequence by Watson-Crick base pairing. The presence of a protospacer-adjacent motif (PAM) directly downstream from the target DNA is a prerequisite for DNA cleavage by Cas9. Cas9 and the sgRNA form a dual endonuclease complex capable of binding the complementary strand of the target site and creating a double-stranded break (DSB) three bases upstream of the PAM. **(B)** In the multiplex CRISPR-Cas9 system for multiple virus control, tandemly arrayed tRNA-sgRNA units each contain a protospacer that is designed from different CMGs: ACMV (AC), EACMCV (EA), and SACMV (SC), and a conserved tracrRNA. The tRNA containing box A and B elements is shown as round rectangles (Xie et al., 2015).

Clustered regularly interspaced short palindromic repeats are specific regions in some bacterial and archaeal genomes that, together with CRISPR associated endonucleases, function as an adaptive immune system for these organisms (see reviews Barrangou and Marraffini, 2014; Barrangou, 2015). The CRISPR system consists of tandem arrays of short, direct repeat sequences, which are separated by ∼20-nt spacer sequences (protospacers) that match the target sequences. The protospacers guide the effector endonucleases (e.g., CRISPR associated, Cas) to target invading nucleic acids based on sequence complementarity. The ease of deployment of this system in genome editing is due to its dependence on the RNA protospacer (Sander and Joung, 2014; Sternberg and Doudna, 2015; Wright et al., 2016). That the CRISPR-Cas system functions efficiently in eukaryotic systems has revolutionized genome editing in plants, and the *Streptococcus pyogenes* endonuclease Cas9 (*Sp*Cas9) has been harnessed for efficient genome editing and gene regulation (Haurwitz et al., 2010; Cong et al., 2013; Jinek et al., 2013) (**Figure 5A**). In this system, Cas9 creates double-strand breaks (DSBs), leading to insertion or deletion (indel) mutations in the targeted gene (Ran et al., 2013). The repair may be by non-homologous end joining (NHEJ) or by homology directed repair (HDR). To simplify engineering and target specificity of the CRISPR-Cas9 system for broad applications, the repeat sequences and protospacers have been combined into a single chimeric guide RNA molecule that is functionally expressed under small nuclear RNA promoters such as U6 or U3 (Cong et al., 2013; Jinek et al., 2013).

Several studies have shown the successful application of the CRISPR-Cas9 system in engineering resistance to geminiviruses. For example, a CRISPR-Cas9 strategy in which guide sequences were designed from the *AC1*, *AV1*, and CRs of *Tomato yellow leaf curl begomovirus* (TYLCV) and *Beet curly top curtovirus* (BCTV) conferred resistance to the respective viruses in *N. benthamiana* plants expressing Cas9 in a virus-specific manner (Ali et al., 2015b). Further, a single sgRNA containing the invariant TAATATTAC nonanucleotide sequence found in the origins of replication of all geminiviruses, targeted and degraded TYLCV, BCTV, and *Merremia mosaic begomovirus*, indicating that this strategy can be used for broad spectrum resistance against geminiviruses. Accordingly, a CRISPR-Cas9 containing sgRNAs designed from diverse coding and non-coding regions of *Beet severe curly top curtovirus* (BSCTV), was shown to specifically target and degrade BSCTV in transgenic *Arabidopsis* and *N. benthamiana* (Ji et al., 2015). Similar results were obtained by Baltes et al. (2015), who designed sgRNAs from the *Bean yellow dwarf mastrevirus* (BeYDV) Rep-binding site, hairpin, nonanucleotide sequence, and three Rep motifs essential for rolling circle replication. This report further showed that sgRNAs targeting sequences near the BeYDV hairpin were less effective, presumably due to formation of secondary structures that impeded Cas9-sgRNA cleavage or access (Baltes et al., 2015). Together, these data demonstrated the potency of the CRISPR-Cas9 system in geminivirus control and therefore will be a good platform to design CMG control. It is important to stress that in addition to directly targeting viral genomes, CRISPR-Cas9 can equally be used to edit host genes that negatively regulate host resistance to virus infection. Thus, current progress in ultra-deep sequencing and the ability to analyze changes in gene expression during virus infection have made it increasingly easy to identify key genes involved in host resistance to virus infection. This has therefore opened up new opportunities to generate host resistance to virus infection given the efficiency of CRISPR to knockout genes.

There is a practical advantage of the CRISPR-Cas9 system over RNAi approaches to control virus infection. For instance, in RNAi (hpRNA, tasiRNA, amiRNA), expression of target genes is mostly repressed, whereas CRISPR-Cas9 knocks out the target genes. This potentially makes CRISPR-Cas9 a very efficient method to generate host resistance. However, it must be noted that an advantage of RNAi approaches over the Cas9 system is that in RNAi the guide RNAs can be extracted directly from invading nucleic acids, thus the capacity for mutational evasion by the virus is limited. In contrast, in the CRISPR-Cas9 platform, sequence determinants are encoded in the host genome, and thus targets have a greater potential for mutational evasion by evolving new virus strains.

A considerable disadvantage of the CRISPR-Cas9 system has been the inability to multiplex the system to simultaneously knock out multiple targets. This limitation has at least partially been resolved by the recent demonstration of engineering of a single polycistronic gene based on the endogenous tRNA-processing system, which was used to generate several sgRNAs (Xie et al., 2015). In this approach, tandemly arrayed tRNA-sgRNA units each contained a conserved tRNA and a sgRNA that includes a 20-nt target-specific spacer (**Figure 5B**). Upon transcription, the tandemly arrayed tRNA-sgRNA chimera is cleaved by endogenous RNase P and RNase Z, releasing a mature sgRNAs, which direct Cas9 to multiple targets (Xie et al., 2015). Because tRNAs and their processing system are conserved in virtually all living organisms, this method can be used to simultaneously target multiple genes in many plant families (Xie et al., 2015). Thus, the multiplex CRISPR-Cas9 platform presents an excellent opportunity to employ a single transgene to simultaneously target multiple CMGs using guide sequences from different viruses.

### CRISPR-Cpf1

Another addition to the CRISPR system toolbox is the recently described *Francisella* spp. CRISPR endonuclease, Cpf1, which has been shown to exhibit features that are not found in Cas9 (Zetsche et al., 2015). Cpf1 recognizes a thymine rich (TTTN) PAM sequence at the 5′ end of the target site, compared with Cas9 PAM (NGG) that is at the 3′ end of the recognition site. CRISPR-Cpf1 was shown to be more efficient and specific than CRISPR-Cas9 in cleaving targets in human cells (Kleinstiver et al., 2016) and has several specific advantages over CRISPR-Cas9 (Lowder et al., 2016). Firstly, Cpf1 does not require tracrRNA and thus the guide RNAs are only 42-nt instead of the ∼100-nt for Cas9, offering cheaper and simpler guide RNA production. Secondly, Cas9-mediated NHEJ usually destroys the PAM site due to PAM proximity to the cleavage site and this prevents future edits. In contrast, Cpf1 cleaves relatively far from the PAM and NHEJ would be less likely to disrupt it, thus ensuring the continued presence of the PAM and cleavage of the target. Thirdly, whereas Cas9 generates blunt ends after cleavage, Cpf1 cleaves in a staggered fashion, creating a 4–5 nucleotide 5′ overhang, thus allowing for directional gene transfer during homologous recombination or HDR. Importantly, because of the relative ease of inserting genes, CRISPR-Cpf1 can be used to introduce virus-resistance amiRNAs, tasiRNAs or other RNA silencing cassettes in predetermined genome loci so as to improve the overall expression and performance of the transgene.

Although there have been numerous reports of efficient application of the CRISPR-Cpf1 approach in mammalian systems since its first description in late 2015, so far only two studies have confirmed its functionality in plant systems. In one study in rice, crRNAs were designed as direct repeats with the potential to target *phytoene desaturase* (*OsPDS*) and *bentazon sensitive lethal* (*OsBEL*) genes. Results showed that the CRISPR-Cpf1 efficiently generated specific and heritable targeted mutations in rice (Xu et al., 2016). Similarly, this approach was used to mutate *phytoene desaturase (PDS*) and *STENOFOLIA* ortholog in *N. tabacum (NtSTF1*) in tobacco, and *Drooping leaf (DL*) and *Acetolactate synthase (ALS*) in rice, respectively (Endo et al., 2016). Hence, because of its inherent advantages over CRISPR-Cas9 system, the CRISPR-Cpf1 system will likely be a formidable tool in plant improvement and should be useful in generating durable resistance to CMGs.

### Non-transgenic CRISPR System

There are new opportunities to transiently edit genes using a non-transgenic CRISPR. In this regard, preassembled Cas9 protein-sgRNA ribonucleoproteins, rather than plasmids that encode them, were used to induce targeted genome modifications in different plant species, and the mutations induced were stably maintained in whole regenerated plants (Woo et al., 2015). Furthermore, Sauer et al. (2016) efficiently generated from edited protoplasts, fertile non-transgenic flax plants with precise genome edits in each of the two flax *ENOLPYRUVYLSHIKIMATE-3-PHOSPHATE SYNTHASE* (*EPSPS*) genes. In this case, the CRISPR components were transfected to flax protoplasts to introduce double-strand breaks without integration into the genome. Because no DNA is transferred, the resulting genome-edited plants might be exempt from current genetically modified organism (GMO) regulations (Kanchiswamy et al., 2015).

In yet another non-transgenic approach, RNA virus-based expression vectors can be engineered to deliver the endonuclease and sgRNAs to induce mutations in a GMO-free manner. Since the viral vector does not integrate into the plant genome, constructs are not transmitted through the germline to the next generation, thus, the induced mutation is free of transgenes. This approach is exemplified with the *Tobacco rattle tobravirus* (TRV)-based vector, which was used to deliver sgRNA targeted to the *PDS* gene in *N. benthamiana* (Ali et al., 2015a). A CaLCV vector has also be used to express sgRNAs in stable Cas9 overexpressing lines of tobacco and was shown to be highly effective at inducing systemic infection and CRISPR-mediated mutagenesis (Yin et al., 2015). In both of these viral delivery examples, the host stably expressed Cas9. However, the possibility to transiently express both sgRNA and the endonuclease exists, so as to obtain a non-transgenic mutant plant. Undoubtedly, future improvements in the non-transgenic gene editing via the CRISPR platform will accelerate cassava genome editing, not only for virus resistance, but also to address other challenges facing the cassava crop.

## Regulations of Genetically Modified (GM) Crops in Developing Countries

During the last two decades, there has been considerable progress in engineering crops to improve yield and quality. However, acceptance of GM technologies will continue to be a challenge in spite of multiple and diverse constraints facing crop production worldwide, including drought, low-yielding crop varieties, pests and diseases, poor soils and dependence on rain-fed agriculture. These problems could all potentially benefit from the application of GM technologies. Moreover, these production constraints are likely to worsen due to a changing climate. Yet, to date, in all of Africa, only South Africa, Egypt, Burkina Faso and recently Sudan produce GM crops, and these are limited to industrial cotton, maize, and soybean. Recently, Malawi, Kenya, Uganda, Nigeria, and Ghana started confined field trials of several GM crops (Adenle, 2011; Black et al., 2011; Okeno et al., 2013; Sinebo and Maredia, 2016); these field trials include CMG and CBSV resistance testing in Kenya, Nigeria, and Uganda (Yadav et al., 2011; Ogwok et al., 2012; Odipio et al., 2014).

The slow rate at which GM technology is being adopted in developing countries can be explained by outright hostility toward GMOs, absence of regulatory policies or lack of resources to implement such policies where they exist. Unfortunately, most of the arguments against GM technology, especially in Africa, are shaped mainly from Europe where the challenges facing agricultural production are less severe and there is a luxury of food choice (Adenle, 2011; Black et al., 2011; Bawa and Anilakumar, 2013). For progress to be made in the development and use of GM technology in developing countries, it is crucial that more efforts be dedicated to educating stakeholders, not only about GM crops in general, but more importantly, about specific technologies developed. For example, much of the current debates on GM technologies have focused on the potential risks associated with transgene flow from GM crops to wild relatives, leading to an imbalance in the ecosystem. While in some instances this is a possibility, such risks do not apply to all crops.

Interestingly, advances in DNA sequencing capabilities have led to the discovery that genetic transformations are a natural occurrence and that plant genomes contain viral genome sequences (Staginnus and Richert-Pöggeler, 2006; Chiba et al., 2011; Chabannes and Iskra-Caruana, 2013; da Fonseca et al., 2016). Thus, many water yam species (*Dioscorea* spp.) have been found to contain transcriptionally active endogenous geminiviral sequences that may be functionally expressed (Filloux et al., 2015). Correspondingly, multiple copies of geminiviral DNA were found in the genomes of four closely related *Nicotiana* spp. suggesting a unique integration event (Ashby et al., 1997). Indeed, the presence of endogenous non-retroviral elements (ENRE) in plant genomes has been suggested to confer viral resistance (Bertsch et al., 2009; Flegel, 2009; Taylor and Bruenn, 2009; da Fonseca et al., 2016). While the functional and evolutionary relevance of these recombination events are yet to be determined, the possibility that integration of a viral genome might have had a functional role (e.g., immunity) in plant genome evolution cannot be ruled out. Such would be analogous to the CRISPR-Cas system where bacteria and archaea copy infecting viral sequences to immunize themselves against future infections (Horvath and Barrangou, 2010).

It is clear that education and involvement of all stakeholders in designing GM technologies is invaluable if the benefits of these technologies are to be exploited for crop improvement, especially in developing countries. To this end, the Virus Resistant Cassava for Africa (VIRCA) (Taylor et al., 2012) project at the Danforth Plant Science Center (St. Louis, MO, USA) recently organized a workshop to assess the risks and consequences of gene flow from transgenic cassava containing CBSV resistance to wild relatives. Based on existing information, this forum concluded that although gene flow is likely to occur, there is only a very small chance that this could lead to a reduction of genetic diversity in the germplasm pool (Hokanson et al., 2016). Similar conclusions would apply to genetically engineered CMG resistance since both approaches involve inserting small viral genome segments into the plant genome. A critically important factor in arriving at this conclusion is the fact that cassava is propagated almost exclusively clonally.

Together, the risks associated with transgenic cassava, a crop that has a low fertility, seed set, and germination rates are largely outweighed by the devastation caused by viruses in cassava growing regions of the world. Moreover, approaches being used in generating resistance to viruses depend on viral genomic sequences, which in some instances integrate naturally in the plant genome or occur episomally, and have been consumed by humans for centuries with no apparent ill effects.

## Conclusion

Cassava is a vital source of food and income in most tropical regions of the world and its production needs to continue to respond to food priorities of developing countries as well as to trends of a global economy. To counter cassava production constraints so as to increase yield and quality, new technologies will need to be deployed. CMD is the most important constraint to cassava production in sub-Saharan African and the Indian subcontinent. To date, introgression of CMD resistance from wild cassava has been by far the most successful CMD control strategy. Three types of CMD resistance loci, designated CMD1, CMD2, and CMD3, have been established; as a precautionary measure against the real possibility of CMD appearing on the American continent, these resistances are being introgressed into South American varieties (Okogbenin et al., 2012). In spite of the important role breeding has played so far in controlling CMGs, it has many challenges, including the length of time required to release a new variety and loss of preferred agronomic attributes. Because of the emergence of new and more virulent CMG species as well as frequent occurrences of mixed infections, which break resistances (**Figure 1D**), new technologies will need to be deployed where breeding is unlikely to succeed. Most genetic engineering strategies depend on use of viral sequences to confer resistance and thus would be helpful in situations where there either is no natural resistance (as apparently is the case with CBSD), or the resistance is difficult to introduce through breeding.

In the last two decades, a lot of progress has been made in identifying and understanding mechanisms of host resistance to virus infections, including particularly RNA silencing and CRISPR systems. As genome sequencing costs fall and new tools are developed to more efficiently analyze genome sequence data, new resistance factors and pathways will continue to be discovered, this would provide additional new tools for use in engineering crops for virus resistance. For example, identification of virus-resistance genes would provide opportunities for such genes to be precisely edited using non-transgenic strategies. Based on the characteristics of the recently discovered CRISPR-Cpf1 system, this approach will likely provide unprecedented new opportunities in cassava improvement through precise transient genome editing, especially given that cassava is recalcitrant to transformation.

## Author Contributions

The author confirms being the sole contributor of this work and approved it for publication.

## Conflict of Interest Statement

The author declares that the research was conducted in the absence of any commercial or financial relationships that could be construed as a potential conflict of interest.
